# Wolbachia-Based Approaches to Controlling Mosquito-Borne Viral Threats: Innovations, AI Integration, and Future Directions in the Context of Climate Change

**DOI:** 10.3390/v16121868

**Published:** 2024-11-30

**Authors:** Francesco Branda, Eleonora Cella, Fabio Scarpa, Svetoslav Nanev Slavov, Annamaria Bevivino, Riccardo Moretti, Abate Lemlem Degafu, Leandro Pecchia, Alberto Rizzo, Francesco Defilippo, Ana Moreno, Giancarlo Ceccarelli, Luiz Carlos Junior Alcantara, Alvaro Ferreira, Massimo Ciccozzi, Marta Giovanetti

**Affiliations:** 1 Unit of Medical Statistics and Molecular Epidemiology, University of Campus Bio-Medico di Roma, 00128 Rome, Italy; f.branda@unicampus.it (F.B.); m.ciccozzi@unicampus.it (M.C.); 2 Burnett School of Biomedical Sciences, College of Medicine, University of Central Florida, Orlando, FL 32827, USA; eleonora.cella@yahoo.it; 3 Department of Biomedical Sciences, University of Sassari, 07100 Sassari, Italy; fscarpa@uniss.it; 4 Butantan Institute, São Paulo 21040-900, Brazil; svetoslav.slavov@fundacaobutantan.org.br; 5 Department for Sustainability, Italian National Agency for New Technologies, Energy and Sustainable Economic Development, ENEA, 00123 Rome, Italy; annamaria.bevivino@enea.com (A.B.); riccardo.moretti@enea.com (R.M.); 6 Unit of Intelligent Health Technologies, Sustainable Design Management and Assessment, Department of Engineering, Università Campus Bio-Medico di Roma, 00128 Rome, Italy; l.abate@unicampus.it (A.L.D.); leandro.pecchia@unicampus.it (L.P.); 7 Laboratory of Clinical Microbiology, Virology and Bioemergencies, Ospedale Sacco, 20157 Milan, Italy; rizzo.alberto@asst-fbf-sacco.it; 8 Istituto Zooprofilattico Sperimentale della Lombardia e dell’Emilia Romagna “B. Ubertini” (IZSLER), 25124 Brescia, Italy; anamaria.morenomartin@izsler.it; 9 Infectious Diseases Department, Azienda Ospedaliero Universitaria Policlinico Umberto I, 00161 Rome, Italy; giancarlo.ceccarelli@uniroma1.it; 10 Mosquitos Vetores: Endossimbiontes e Interação Patógeno-Vetor, Instituto René Rachou-Fiocruz, Belo Horizonte 30190-002, Brazil; luiz.alcantara@ioc.fiocruz.br (L.C.J.A.); alvaro.ferreira@ioc.fiocruz.br (A.F.); 11 Department of Sciences and Technologies for Sustainable Development and One Health, Universita Campus Bio-Medico di Roma, 00128 Rome, Italy; 12 Oswaldo Cruz Institute, Oswaldo Cruz Foundation, Rio de Janeiro 21040-900, Brazil

**Keywords:** *Wolbachia*, mosquito control, vector-borne diseases, climate change, public health

## Abstract

Wolbachia-based mosquito control strategies have gained significant attention as a sustainable approach to reduce the transmission of vector-borne diseases such as dengue, Zika, and chikungunya. These endosymbiotic bacteria can limit the ability of mosquitoes to transmit pathogens, offering a promising alternative to traditional chemical-based interventions. With the growing impact of climate change on mosquito population dynamics and disease transmission, Wolbachia interventions represent an adaptable and resilient strategy for mitigating the public health burden of vector-borne diseases. Changes in temperature, humidity, and rainfall patterns can alter mosquito breeding habitats and extend the geographical range of disease vectors, increasing the urgency for effective control measures. This review highlights innovations in Wolbachia-based mosquito control and explores future directions in the context of climate change. It emphasizes the integration of Wolbachia with other biological approaches and the need for multidisciplinary efforts to address climate-amplified disease risks. As ecosystems shift, Wolbachia interventions could be crucial in reducing mosquito-borne diseases, especially in vulnerable regions. AI integration in Wolbachia research presents opportunities to enhance mosquito control strategies by modeling ecological data, predicting mosquito dynamics, and optimizing intervention outcomes. Key areas include refining release strategies, real-time monitoring, and scaling interventions. Future opportunities lie in advancing AI-driven approaches for integrating Wolbachia with other vector control measures, promoting adaptive, data-driven responses to climate-amplified disease transmission.

## 1. Introduction

*Wolbachia* spp. are a genus of intracellular alpha-proteobacteria widely distributed among arthropods, including mosquitoes and some parasitic nematodes [1]. These bacteria are primarily transmitted vertically, from imago to larvae passing from the ovaries to the oocytes, but they can also be transmitted horizontally between species, especially among closely related hosts [2], by predator–prey [3] or host–parasitoid interactions [4], by shared plants [5] or other food resources [6], and by kleptoparasitism [7]. Discovered nearly a century ago, Wolbachia has since been identified as one of the most widespread endosymbiotic bacteria on Earth, infecting a vast array of insect species. The influence of Wolbachia on its hosts is multifaceted, ranging from reproductive manipulations, such as cytoplasmic incompatibility, parthenogenesis, and male-killing, to mutualistic relationships that enhance host survival and reproduction [8]. In mosquitoes, Wolbachia’s ability to manipulate reproduction and reduce the transmission of vector-borne pathogens has garnered significant attention as a sustainable and innovative approach to disease control [8]. By interfering with pathogen development within the mosquito, *Wolbachia*-based interventions are less capable of transmitting viruses such as dengue, Zika, and chikungunya to humans [9]. This has led to the development of Wolbachia-based interventions, where populations of infected mosquitoes are introduced into the environment to either suppress or replace wild mosquito populations [9]. As global climate change continues to alter ecosystems, including mosquito habitats, the dynamics of vector-borne disease transmission are changing. Warmer temperatures, increased rainfall, and shifting humidity patterns can expand the geographical range of mosquitoes, leading to the emergence of diseases in previously unaffected regions [10]. Wolbachia-based strategies offer a promising solution to this growing public health challenge, providing a biologically adaptive method for reducing mosquito populations and their ability to transmit pathogens. This review delves into the current applications of Wolbachia in mosquito control and explores future directions, particularly in the context of climate change and its role in exacerbating mosquito-borne diseases. By integrating Wolbachia-based strategies with AI, ecological, and climate data, we can better understand how to optimize this approach in an evolving global landscape.

## 2. Wolbachia Strain and Mosquito Infection

**Figure 1:**
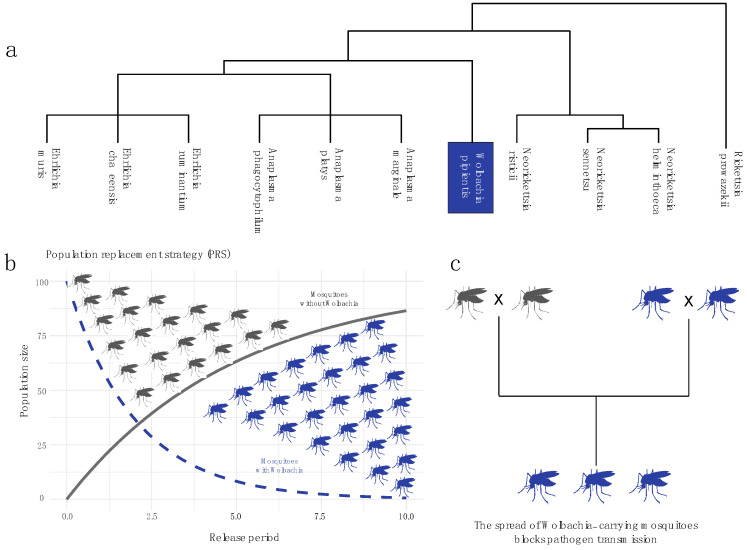
**Wolbachia-based population replacement strategy for mosquito-borne disease control.** (**a**) Phylogenetic relationship of *Wolbachia pipientis* with other members of the *Anaplasmataceae* family, showing its evolutionary ties to other intracellular bacterial pathogens; (**b**) Population replacement strategy (PRS) graph, illustrating how the introduction of *Wolbachia*-based interventions can reduce the population of uninfected individuals over time. The graph shows a projected decrease in the population size of uninfected mosquitoes (dashed blue line) and the concurrent rise of *Wolbachia*-based interventions (solid gray line) as a function of the release period; (**c**) Mechanism of transmission blocking, where the spread of mosquitoes infected by *Wolbachia* strains capable of interfering with pathogen replication effectively blocks pathogen transmission and reduces the spread of mosquito-borne diseases. In panels (**b**,**c**), blue mosquitoes represent *Wolbachia*-infected individuals, while gray mosquitoes represent uninfected individuals.

*Wolbachia* is a genus of Gram-negative bacteria acting as endosymbiotic bacteria living in many orders of insects and in other invertebrates [11,12]. It belongs to *Alphaproteobacteria* (Rickettsiales: Anaplasmataceae) and is predicted to infect more than 40% of insect species [13,14]. The type species for the *Wolbachia* genus is *Wolbachia pipientis*, which was first described in the mosquito *Culex pipiens* [15]. The related genera *Anaplasma*, *Ehrlichia*, and *Rickettsia* typically have life cycles involving both an invertebrate vector and a mammalian host, though some species are exclusively associated with invertebrates [16] (Figure 1a). Phylogenetic analysis shows their close relationship with other intracellular bacterial pathogens. However, unlike these related genera, *Wolbachia* does not infect vertebrates. *Wolbachia* lives exclusively within host cells and spreads by altering the biology of its host species [17]. Its primary transmission occurs through vertical inheritance via the maternal cytoplasm, though horizontal transmission between insect species also contributes to its prevalence [18,19,20,21,22]. *Wolbachia Wolbachia* typically invades invertebrate populations by conferring reproductive or fitness advantages to infected individuals, and under favorable conditions this invasion can reach high prevalence within 1–2 years [23]. After its release period it is possible to observe a complete population replacement (Figure 1b). The potential use of *Wolbachia* in controlling mosquito-borne diseases has gained attention as an environmentally friendly and cost-effective alternative to insecticide-based methods. *Wolbachia*-induced cytoplasmic incompatibility (CI) was first proposed for *Culex* mosquito control in 1967 [24] with trials conducted in India in the 1970s [25]. CI is a common reproductive manipulation that increases the proportion of *Wolbachia*-infected individuals in a population. *Wolbachia*-infected females can mate with either uninfected males or males infected with the same or a compatible *Wolbachia* strain [26] (Figure 1c). CI occurs when a *Wolbachia*-infected male mates with a female that is either uninfected (unidirectional CI) or infected with an incompatible strain (bidirectional CI) [27]. Essentially, if the male carries a Wolbachia strain that is not present in his mate, the cross is incompatible [28].

CI can also be exploited to favor the spread of *Wolbachia* strains capable of reducing the vector competence of suitable mosquito vectors. *Wolbachia* can affect viruses in two ways: by reducing or delaying virus accumulation, and by decreasing or delaying virus-induced host mortality. While many factors influence vector competence for transmitting arboviruses in mosquitoes, the presence of *Wolbachia* may alter this competence by affecting the mosquito’s susceptibility to viral infection [29,30,31,32]. After its discovery in *Culex pipiens* [30], this endosymbiont has been found to be naturally harbored by various wild mosquito populations including species that transmit different pathogens to humans [33]:

i. *Aedes aegypti*: carries viruses such as dengue, chikungunya, and Zika, as well as nematodes such as filarial and mermithid [34].
ii. *Aedes albopictus*: transmits viruses including dengue, chikungunya, Eastern equine encephalitis, La Crosse, Zika virus, Venezuelan equine encephalitis virus and West Nile, and Japanese encephalitis, along with filarial and mermithid nematodes [35].
iii. *Aedes polynesiensis*: spreads viruses such as Zika, dengue, and Ross River, along with filarial nematodes [36].
iv. *Culex pipiens* species complex: vectors viruses such as West Nile, Usutu virus, Sindbis, Eastern equine encephalitis, Venezuelan equine encephalitis, Japanese encephalitis, St. Louis encephalitis, Ross River, Murray Valley encephalitis, and Rift Valley fever, as well as filarial nematodes [35,37,38].
v. *Anopheles stephensi*: responsible for transmitting malaria parasites and O’nyong-nyong virus [39].

There are many factors that determine vector competence for the transmission of arboviruses in mosquitoes [29], but the presence of *Wolbachia* could influence this by altering the mosquito’s susceptibility to viral infection. Studies have shown that naturally occurring *Wolbachia* was present in 7–42% of *Culex* species analyzed, 0–30% of *Aedes* species, and 1–15% of *Anopheles* species [40,41,42,43]. Notably, *Wolbachia* is frequently detected in common arbovirus vectors such as the *Culex pipiens* complex and *Aedes* species, including *Ae. albopictus*, but not in *Ae. aegypti*. The establishment of *Wolbachia* in certain mosquito species can be hindered by the native microbiome, which may explain its absence in some species [44]. However, the artificial horizontal transfer of certain *Wolbachia* strains in either uninfected or already infected hosts has proven feasible [45]. *Wolbachia* can be used in various ways for disease control. These include the following: (i) reducing vector populations by releasing *Wolbachia*-infected males that are incompatible with females [46]; (ii) introducing Wolbachia strains that cause fitness disadvantages, especially in seasonally variable environments [47]; and (iii) decreasing the ability of vectors to transmit diseases by introducing Wolbachia strains that directly interfere with disease transmission and capability of spreading thanks to CI [48,49]. Wolbachia interferes with viral replication within mosquitoes by inducing an immune response that limits viral replication and competition for resources within host cells [50,51,52]. Field trials have demonstrated the effectiveness of Wolbachia in reducing dengue transmission, such as in areas where Wolbachia-infected *Aedes aegypti* were released [53,54]. There are an enormous diversity of *Wolbachia* strains in nature. *Wolbachia* has a circular chromosome ranging from 1.08 Mb to 1.7 Mb and containing a high number of mobile and repetitive elements [49]. The strains *w*Mel and *w*MelPop *w*MelCS are naturally found in D. melanogaster, *w*Au in D. simulans, and *w*Inn in D. innubila. The strain *w*Ri is native to D. simulans, while *w*AlbB is present in Ae. albopictus, and *w*Pip is found in the Culex pipiens complex [17]. The mosquito species *Ae. albopictus*, *Ae. polynesiensis*, and *Ae. aegypti* have been stably transfected with one or more of three *Wolbachia* strains including *w*Mel, *w*MelPop, *w*AlbB, and *w*Pip [55,56]. Despite these successes, challenges remain, such as the potential for resistance development in mosquito populations and the ecological impacts of releasing Wolbachia-infected mosquitoes. Future research may focus on new techniques for more efficient Wolbachia transfer and combining Wolbachia with other vector control strategies [57,58].

## 3. The Impact of Climate Change on Mosquito-Borne Disease Emergence

**Figure 2:**
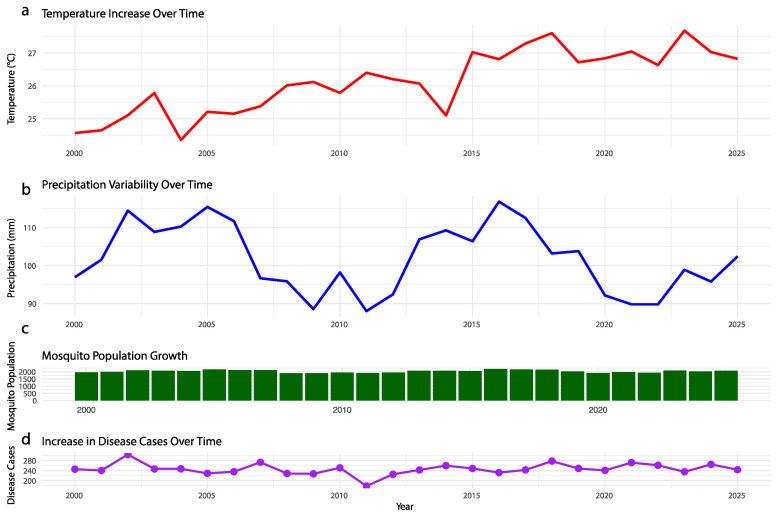
**The impact of climate change on mosquito-borne disease transmission dynamics.** This figure presents a model-based simulation rather than real-world data. It illustrates (**a**) temperature increase over time (2000–2025), showing a gradual rise in global average temperatures, which can extend mosquito activity and viral transmission periods; (**b**) precipitation variability over time, illustrating fluctuations in rainfall patterns that influence mosquito breeding habitat availability; (**c**) mosquito population growth, representing the projected increase in mosquito populations driven by climatic changes over the same period; (**d**) increase in disease cases over time, correlating the rise in global temperatures and altered precipitation patterns with the increase in reported mosquito-borne disease cases worldwide.

Climate change is increasingly recognized as a critical driver of mosquito-borne disease emergence and resurgence globally [59]. Rising global temperatures, altered precipitation patterns, and the increased frequency of extreme weather events are fundamentally changing the distribution, abundance, and breeding cycles of mosquito vectors (Figure 2).

Temperature increases can accelerate mosquito spread, shorten viral incubation periods, and extend transmission seasons, particularly in regions previously unsuitable for vector survival [60]. Additionally, variability in precipitation affects the availability of mosquito breeding sites, as both excessive rainfall and drought can create conditions favorable for mosquito proliferation [60]. Consequently, viral fevers such as dengue, chikungunya, and West Nile virus are expanding into new geographic areas, highlighting the growing need for enhanced surveillance and control strategies. These environmental shifts have also facilitated the spread of viruses into new regions, creating conditions conducive to the emergence of diseases such as those determined by Zika virus (ZIKV), dengue virus (DENV), chikungunya virus (CHIKV), yellow fever virus (YFV), and Oropouche virus (OROV) [61,62,63,64,65]. Focusing on these pathogens, rather than others, reflects their significant epidemiological impact and their strong association with climate-sensitive mosquito vectors. ZIKV, a member of the Flaviviridae family, gained global attention during the 2015–2016 outbreaks in South America, particularly in Brazil [65]. Before this, ZIKV had caused sporadic outbreaks in Africa and Southeast Asia, but climate change, particularly rising temperatures and altered precipitation patterns, played a crucial role in expanding the habitat of Aedes aegypti, ZIKV’s primary vector, into new regions [65]. The severe clinical picture of ZIKV, especially in neonates linked to microcephaly and other birth defects, underscored the urgent need for adaptive strategies to mitigate the effects of climate change on vector-borne diseases [66]. Similarly, dengue virus, also in the *Flaviviridae* family, has experienced a resurgence in regions where the disease was previously controlled or where it was once rare or nonexistent. DENV is the most widespread mosquito-borne virus, exposing billions of people at risk [67]. Climate change contributes to the resurgence of dengue by enhancing mosquito survival rates and shortening the time required for the viral life cycle in mosquitoes [68]. Rainfall variability also creates ideal breeding conditions for *Ae. aegypti* and *Ae. albopictus*, the primary vectors of dengue, through periods of heavy rainfalls followed by droughts. Recent outbreaks have occurred in temperate regions, such as southern Europe and parts of the United States, where dengue was previously uncommon, highlighting the role of global warming in driving the northward expansion of vector populations [67]. CHIKV, a member of the *Togaviridae* family, emerged as a global health threat in 2004, spreading beyond its endemic areas in Africa and Asia to cause large outbreaks in the Indian Ocean islands, Europe, and the Americas [61]. The rapid spread of CHIKV is strongly linked to the adaptability of *Aedes aegypti* and *Aedes albopictus*, which thrive in new climatic conditions [69]. The virus has now become endemic in parts of the Americas, leading to regular outbreaks in regions where it was once unknown [69]. YFV, another Flaviviridae member, has experienced significant re-emergence in parts of Africa and South America [56]. Historically controlled by vaccination, YFV is making a resurgence due to weakened health infrastructures, population growth, deforestation, and climate change [64]. The increasing range of *Aedes aegypti*, driven by rising temperatures and changing rainfall patterns, has allowed YFV to resurge in regions such as Brazil, with outbreaks extending beyond the Amazon basin into areas with no recent history of yellow fever transmission [64]. These outbreaks, often in densely populated urban centers, underscore the importance of addressing climate change as a driver of mosquito-borne disease emergence, particularly in vulnerable regions with limited healthcare access [64]. OROV, a member of the *Peribunyaviridae* family, is a neglected mosquito-borne pathogen historically confined to the Amazon basin [63]. Recently, however, OROV has expanded beyond the Amazon, spreading into new regions of South America, including urbanized areas [63]. OROV is primarily transmitted by the *Culicoides paraensis* midge, but there are concerns about its potential to be transmitted by *Culex* mosquitoes. Climate change, including warmer temperatures, habitat destruction, and changing rainfall patterns, is likely driving the vector’s movement into new ecosystems, contributing to OROV’s rapid epidemic spread. This rapid expansion highlights the need for robust surveillance and control measures, as OROV poses a significant public health threat with the potential for large urban outbreaks in previously unaffected areas [63]. The geographical range of *Aedes aegypti* and *Aedes albopictus* is expanding northward due to global warming. Historically confined to tropical and subtropical regions, these mosquitoes are now establishing populations in temperate zones, such as parts of Europe and North America [70]. Additionally, research has demonstrated that *Wolbachia* infections in mosquitoes are sensitive to temperature variations, which can influence *Wolbachia* density, transmission efficiency, and its pathogen-blocking capacity. For instance, studies indicate that fluctuating temperatures can impact the rate of DENV infection in mosquitoes and their ability to transmit the virus, as seen in settings where temperature oscillates around a mean. Despite these fluctuations, *Wolbachia* has shown a consistent ability to reduce DENV infection and transmission potential across various temperature ranges, supporting its robustness as a biocontrol tool in certain environments, such as those in Cairns, Australia [71]. However, these findings underscore the importance of evaluating *Wolbachia* efficacy under field-relevant temperature conditions for different regions, as warmer or more variable climates may alter outcomes. A more comprehensive approach that considers the local environmental factors and the variability in *Wolbachia* performance across different temperature regimes will help clarify how climate change could influence mosquito-borne disease risks.

## 4. The Role of Artificial Intelligence in *Wolbachia* Research and Future Challenges

Artificial intelligence (AI) is rapidly transforming various fields of science and technology, including biological research [72]. AI’s capacity to analyze large datasets, identify complex patterns, and make accurate predictions enhances our understanding of intricate biological systems, such as the relationship between *Wolbachia* and its arthropod hosts [72]. AI might play a central role in three key areas of Wolbachia research: genomic data analysis, modeling host–*Wolbachia* interactions, and ecological and evolutionary modeling (Table 1).

In genomic analysis, AI enables the rapid and precise identification of genetic variants, functional annotation of genes, and prediction of protein–protein interactions, significantly accelerating the understanding of *Wolbachia*’s molecular biology [73]. Regarding host–*Wolbachia* interaction modeling, AI provides tools to simulate and predict *Wolbachia*’s effects on host physiology, behavior, and fitness. These models offer valuable insights into mechanisms such as reproductive manipulation, cytoplasmic incompatibility, and *Wolbachia*-mediated pathogen resistance. In the realm of ecological and evolutionary modeling, AI is revolutionizing predictions of *Wolbachia*’s spread in arthropod populations and its impact on host population dynamics. These models integrate various factors influencing *Wolbachia*–host dynamics, including (i) environmental variables such as temperature, humidity, and resource availability, which affect both *Wolbachia* and its hosts; (ii) ecological interactions, such as competition, predation, and other biotic factors that shape arthropod communities; (iii) evolutionary processes such as mutation rates, natural selection, genetic drift, and gene flow that shape the evolution of *Wolbachia* and its hosts; (iv) specific *Wolbachia* features, including transmission rates, effects on host fitness, and cytoplasmic incompatibility; and (v) anthropogenic factors such as disease control interventions, land-use changes, and other human impacts on ecosystems.

Moreover, AI offers new possibilities for exploring “what-if” scenarios and optimizing intervention strategies. Researchers can use these models to simulate future scenarios, ranging from climate variations to changes in environmental management practices, assessing their impact on *Wolbachia*–host dynamics. As research advances, AI is poised to play an increasingly central role in disease control, not only enhancing our understanding of *Wolbachia*–host interactions but also enabling manipulation of these relationships to develop innovative solutions for environmental sustainability. These advances open new avenues for scientific inquiry, offering creative and sustainable solutions to complex biological challenges.

### 4.1. AI-Driven Genomic Data Analysis

AI has revolutionized genomic data analysis by providing tools capable of handling the complexity and volume of genomic information [74]. AI algorithms can identify patterns and make predictions that traditional statistical methods might overlook. In genomic data analysis, AI has facilitated advances in key areas such as sequence alignment, variant calling, gene expression analysis, and the interpretation of noncoding regions [75]. Sequence alignment, a crucial process for identifying similarities and differences between DNA sequences, has greatly benefited from AI. Deep learning models, including convolutional neural networks (CNNs), have enhanced the accuracy and speed of sequence alignment, reducing computational costs and time. This has proven particularly valuable for large-scale projects in population genomics and personalized medicine. For example, Rakotonirina et al. [75 used Matrix-Assisted Laser Desorption Ionization–Time of Flight (MALDI–TOF) in combination with CNNs to process spectral data and improve the detection of *Wolbachia* in *Aedes aegypti*, increasing the efficiency of identifying infected mosquitoes. In variant calling, AI-driven approaches have transformed the identification of genetic variations. Zhu et al. demonstrated the use of deep learning for refining somatic variant calling in cancer sequencing data, while Singh et al. introduced PEPPER-Margin-DeepVariant, a haplotype-aware variant calling pipeline that produces state-of-the-art results using nanopore data [76]. These innovations highlight the growing role of AI in genomic precision. Gene expression analysis involves measuring the activity of thousands of genes to capture a global picture of cellular function. AI algorithms can efficiently process high-dimensional data from RNA sequencing (RNA-seq) and single-cell RNA sequencing (scRNA-seq), identifying expression patterns that indicate disease states or treatment responses. Techniques such as autoencoders and recurrent neural networks (RNNs) have been employed to impute missing values and classify cells by expression profiles. In the realm of genetic control strategies, Iftikhar et al. explored *Wolbachia*-infected male mosquitoes as a method to suppress *Aedes aegypti* populations. Using AI-supported mathematical models, they accurately forecasted disease control outcomes by simulating different mating scenarios and identifying key parameters critical for implementation. Their findings highlight the importance of theoretical models in designing cost-effective strategies for future experimental applications. AI has also been instrumental in the interpretation of *Wolbachia* toxin–antidote protein functions. Beckmann et al. used evolutionary algorithms to model cytoplasmic incompatibility (CI) systems in insects, focusing on the evolution of toxin–antidote (TA) systems. By simulating protein string evolution, their research provided insights into how nuclear localization signals (NLS) and Type IV secretion system (T4SS) signals influence the evolution of CI mechanisms. Their study offers a framework for understanding the molecular evolution of CI systems in nature. A significant portion of the human genome consists of noncoding regions, which play regulatory roles in gene expression. AI has been essential in predicting the functions of these regions by integrating data on chromatin accessibility, histone modifications, and transcription factor binding sites. For instance, algorithms such as DeepSEA use deep learning to predict the impact of noncoding variants on gene expression and disease phenotypes [77,78]. While extensive studies have been conducted on the gene structures of well-known species such as *E. coli*, fewer resources exist for newly sequenced genomes such as *Wolbachia*. Gene prediction models trained on one species may not accurately reflect the characteristics of other prokaryotic organisms. This challenge was encountered when predicting genes in the *Wolbachia* genome. A neural network-based gene prediction model was developed, using coding sequences as a positive dataset and intergenic regions as a negative dataset. The resilient propagation learning algorithm demonstrated superior performance on a multi-layer perceptron neural network, consisting of 64 input nodes, 10 hidden nodes, and 1 output node. Additionally, Etebari et al. [78] characterized the miRNA profiles in *Aedes aegypti* cells with and without *Wolbachia* infection. They observed a general increase in small RNAs (18–28 nucleotides) in both cell compartments of infected cells, identifying specific miRNAs that were either induced or suppressed by *Wolbachia* infection. The study also revealed changes in piRNA abundance, offering promising insights into host–endosymbiont interactions and how *Wolbachia* manipulates the host miRNA machinery to maintain its replication. MiRanalyzer, a web-based tool using machine learning (Support Vector Machine) to predict new miRNA candidates, was employed to analyze high-throughput sequencing data. The tool demonstrated a high level of accuracy, even when using genomic references from proxy species.

### 4.2. Modeling Host–Wolbachia Interactions

Understanding the interactions between *Wolbachia* and its hosts is essential for developing effective disease control strategies, such as using *Wolbachia*-based interventions to combat dengue and Zika viruses [79]. AI has been instrumental in advancing the modeling of *Wolbachia*–host interactions by allowing researchers to analyze large datasets on infection prevalence, host fitness, and transmission rates. Machine learning models predict how *Wolbachia* spreads, its impact on host reproduction, and potential resistance development. For example, Faiz et al. [80] developed a computational framework using a Bayesian regularization backpropagation neural network (BRB-NN) to model *Wolbachia*-infected and uninfected mosquito populations, accounting for incomplete cytoplasmic incompatibility and imperfect maternal transmission. The model demonstrated how fractional order derivatives and reproduction rates of *Wolbachia*-based interventions affect population dynamics. Another study assessed the effectiveness of releasing *Aedes aegypti* mosquitoes infected with the *wMel* strain in Rio de Janeiro, Brazil [81,82,83]. Over 67 million mosquitoes were released to reduce dengue and chikungunya incidence. While *wMel* presence was lower in areas with high disease prevalence, there was a 38% reduction in dengue cases and 10% in chikungunya. Although *wMel* introgression was not complete, these results suggest even intermediate levels of *Wolbachia* can reduce disease incidence, offering important insights for future release programs.

### 4.3. Ecological and Evolutionary Modeling

AI has additionally revolutionized ecological and evolutionary modeling by providing tools to handle complex, non-linear systems with multiple interacting components. These models are crucial for understanding species interactions, biodiversity, and ecosystem dynamics, particularly in the context of environmental change [84,85]. AI enhances species distribution models (SDMs), which capture ecological niches and predict suitable habitats. However, SDMs show variable predictive success, with better performance for species presence (~53%) than for abundance, population fitness, or genetic diversity. This indicates the need for SDMs to be treated as hypotheses to be tested with independent data, especially in conservation planning [85,86]. AI techniques, such as neural networks and ensemble learning, improve the accuracy of these models by identifying complex relationships between species and their environments [86]. MaxEnt, a machine learning method, has been widely used for species distribution modeling with presence-only data [86]. For instance, a study on the invasive mosquito species *Aedes albopictus* in Pennsylvania used MaxEnt to evaluate environmental and neighborhood factors, achieving a 74.7% accuracy in predicting mosquito presence. This model highlighted how environmental variables explain suburban and rural conditions, while neighborhood factors better predict urban patterns. In addition, AI-based large language models (LLMs) have proven their ability to efficiently manage ecological data, including species distribution, conservation needs, and the invasion of alien species such as *Ae. albopictus*, where manual processing is a significant challenge [86]. On the other hand, van Hoek et al. recently highlighted the potential role of LLM-based agents into prevention and control of infectious disease outbreaks [87,88]. The concomitant deployment of multiple specific LLM-based agents in the context of ecological and epidemiological data might the enhance the efficiency of data management.

AI also plays a crucial role in modeling the effects of climate change on ecosystems. By integrating data from satellite imagery, climate models, and ecological surveys, AI can predict how climate change will alter species distributions, community composition, and ecosystem services. Ogunlade et al. [85] modeled interactions between mosquito populations infected with different *Wolbachia* strains, showing that introducing a single strain with optimal traits—such as high maternal transmission and cytoplasmic incompatibility—may be more effective than using multiple strains. While AI’s potential in ecological and evolutionary modeling is immense, challenges remain, including managing large and heterogeneous datasets, ensuring model interpretability, and addressing ethical considerations in environmental management. Future advancements will require interdisciplinary collaboration and responsible research practices to fully harness AI’s potential in *Wolbachia* research and disease control.

## 5. Safety and Ethical Considerations in the Wolbachia-Based Mosquito Control Strategy

**Figure 3:**
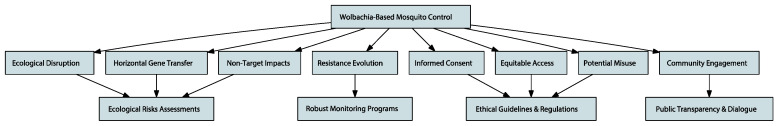
**Key safety and ethical considerations in Wolbachia-based mosquito control.** This diagram outlines the major concerns and necessary precautions surrounding the implementation of Wolbachia-based strategies. The potential risks include ecological disruption, horizontal gene transfer, non-target impacts, and resistance evolution. These risks require proactive ecological risk assessments and robust monitoring programs to ensure safety. Ethical considerations are centered around informed consent, equitable access to the benefits of the intervention, community engagement, and potential misuse. Public transparency and dialogue, alongside the development of clear ethical guidelines and regulations, are essential to addressing these concerns.

While the Wolbachia-based strategy for mosquito control holds significant promise for combating vector-borne diseases, it raises several safety and ethical concerns that warrant careful consideration [89,90,91]. One major safety concern is ecological disruption. It is important to distinguish between two key approaches: the population replacement strategy, which introduces *Wolbachia* into wild mosquito populations, and the Incompatible Insect Technique (IIT), which reduces egg fertility in wild populations without interfering with infection types. In IIT, male mosquitoes infected with *Wolbachia* are released, and since these males are incompatible with wild-type females, no viable offspring are produced. Importantly, their sperm do not harbor *Wolbachia*, thus avoiding horizontal transfer of the bacteria. Introducing *Wolbachia* through the population replacement strategy could have unforeseen ecological consequences. *Wolbachia* can influence mosquito fitness and competitiveness, potentially disrupting existing species interactions, which may impact food webs or lead to the emergence of more resilient vector species [89,90,91] (Figure 3). Therefore, different risk assessments and monitoring strategies are required for each method.

Another safety concern involves horizontal gene transfer. Although unlikely, the possibility of *Wolbachia* genes transferring to other organisms, including mosquitoes or even humans, cannot be entirely ruled out, and such transfers could have unpredictable and potentially harmful consequences [92]. Additionally, there is the risk of the evolution of resistance. As with any intervention targeting a biological organism, the widespread use of *Wolbachia* could drive the evolution of resistance in mosquito populations, potentially rendering the strategy ineffective over time. Unintended impacts on non-target organisms also present a concern. While *Wolbachia*-based interventions primarily target specific disease vectors, there is a risk of unintended impacts on non-target organisms, such as predators that rely on mosquitoes as a food source [82]. In terms of ethical concerns, informed consent is a critical issue. Deploying *Wolbachia*-based interventions on a large scale raises questions about whether communities are fully informed about the potential risks and benefits associated with the intervention. Community engagement is another crucial ethical aspect. The successful implementation of this strategy requires robust community engagement and dialogue to address concerns, build trust, and ensure that the intervention aligns with local values and priorities. Ensuring equitable access to the benefits of this technology, particularly for communities disproportionately burdened by mosquito-borne diseases, is also crucial. Additionally, there is the potential for misuse or unintended consequences, as with many technologies, making it essential to establish safeguards and ethical guidelines to prevent the malicious use of *Wolbachia*-based strategies. Addressing these safety and ethical concerns requires a multifaceted approach. This includes conducting comprehensive ecological risk assessments before and during field trials to evaluate potential impacts on both target and non-target organisms. Implementing robust monitoring programs to track the spread of *Wolbachia*, assess its long-term effects, and detect any unintended consequences is also necessary. Promoting transparency and open communication with the public about the potential risks and benefits of the technology is essential. Developing clear ethical guidelines and regulations governing the research, development, and deployment of *Wolbachia*-based strategies will help ensure the responsible and ethical use of this promising technology for global health.

## 6. Discussion

The integration of Wolbachia-based approaches into mosquito control strategies represents a promising avenue for the mitigation of vector-borne diseases such as dengue, Zika, and chikungunya [93]. As highlighted throughout this review, innovations driven by AI have played a transformative role in advancing genomic data analysis, host–Wolbachia interaction modeling, and ecological and evolutionary predictions [94]. These technological advances have enabled the field to move from conceptual understanding to applied interventions with tangible outcomes, such as reduced disease incidence in areas where *Wolbachia*-based interventions have been released. AI has facilitated new ways to model complex biological systems, allowing researchers to predict *Wolbachia* spread and evaluate its effects on mosquito populations with greater accuracy [81]. In genomic data analysis, AI tools have revolutionized variant calling and gene expression profiling, contributing to our understanding of *Wolbachia*’s molecular biology and its manipulation of host species. This is particularly crucial in a rapidly changing climate, where dynamic environmental factors affect both mosquito and *Wolbachia* fitness. AI-based ecological models, such as those using MaxEnt, have provided more reliable predictions about how *Wolbachia*-based interventions might thrive under different environmental conditions, which is key to long-term success [91]. However, alongside these innovations come significant challenges. As we have seen, the introduction of Wolbachia into natural mosquito populations is not without risk. Ecological disruptions, such as altered species interactions or the emergence of more resilient vectors, cannot be overlooked [90]. Furthermore, Wolbachia infection may not be suitable for all vector species, as some mosquitoes show resistance to infection, and the ability to efficiently mass-rear these mosquitoes under laboratory conditions remains a logistical challenge. These limitations, combined with the financial costs of running large-scale control programs, must also be considered. The potential for horizontal gene transfer, while deemed unlikely, remains a concern with unpredictable consequences. Similarly, the evolution of resistance in mosquito populations poses a long-term risk that could undermine the effectiveness of Wolbachia-based strategies. Continuous monitoring and adaptation will be necessary to address these potential challenges, especially as climate change alters the ecosystems in which these interventions are deployed [92]. Another key issue is the ethical implications of deploying *Wolbachia*-based interventions at scale. Ensuring that communities are informed and engaged throughout the process is critical for maintaining public trust and acceptance. Informed consent and transparency about both the benefits and risks of such interventions are essential. Equitable access to the technology is also important, particularly for regions that are disproportionately affected by mosquito-borne diseases but may lack the resources or infrastructure to implement *Wolbachia*-based programs effectively. These ethical considerations underscore the importance of a socially responsible and inclusive approach to the development and deployment of this technology. Looking forward, the future directions for *Wolbachia*-based mosquito control are closely linked to ongoing advances in AI and related technologies. The ability of AI to process vast amounts of data, model complex systems, and simulate future scenarios will continue to be crucial for optimizing *Wolbachia* deployment strategies. For instance, models that incorporate climate change projections can help to predict how shifts in temperature, precipitation, and habitat availability will impact both mosquitoes and *Wolbachia*, allowing for better planning and adaptation of intervention strategies [93]. AI’s capacity for continuous learning and improvement will be essential in refining these models as new data becomes available. Furthermore, AI can play a pivotal role in the development of more targeted approaches [88]. By integrating genomic, ecological, and environmental data, researchers can refine release strategies to focus on regions where the introduction of *Wolbachia* will have the greatest impact, while minimizing risks to non-target species and ecosystems. For example, future studies could explore the use of AI to predict the optimal release times and locations based on environmental variables, mosquito population dynamics, and human activity patterns, thus maximizing the efficacy of *Wolbachia*-based interventions [94,95]. In addition to technological advancements, it is imperative that researchers and policymakers work together to establish clear ethical guidelines and regulatory frameworks for the use of *Wolbachia*-based strategies. As these interventions become more widespread, the establishment of global standards for risk assessment, monitoring, and public engagement will ensure that the technology is used responsibly and sustainably. The involvement of international organizations, such as the World Health Organization (WHO), in developing these frameworks will be essential for harmonizing efforts across different regions and ensuring that all communities benefit from the advancements in *Wolbachia*-based mosquito control. By balancing scientific ambition with social responsibility, we can harness the full potential of *Wolbachia* to create sustainable, long-term solutions for vector-borne disease control in a rapidly changing world.

## Figures and Tables

**Table 1:** AI applications in *Wolbachia* research, outlining key research areas, challenges, and future opportunities.

*Area of Research*	*AI Application*	*Challenges*	*Future Opportunities*
***Genomic data analysis***	AI for identifying genetic variants, functional annotation, and protein–protein interactions.	Handling complex and large genomic datasets.	Improving prediction accuracy for gene function and expression, especially for noncoding regions.
			Best models effectively capture gene interactions and functions, which can be enhanced by more refined data integration through AI.
***Modeling host–Wolbachia interactions***	AI models for simulating *Wolbachia* effects on host physiology, behavior, and fitness.	Incomplete data on host–microbe interactions, potential resistance development.	Optimizing *Wolbachia*-based intervention strategies for disease control.
			Models that incorporate feedback mechanisms and adaptability are most effective; AI can enhance by improving predictive modeling of host–pathogen interactions.
***Ecological and evolutionary modeling***	AI models to predict *Wolbachia* spread, and its impact on host population dynamics.	Integrating diverse factors such as climate, ecology, and evolution; ensuring interpretability of models.	Modeling “what-if” scenarios for future environmental changes and interventions.
			Models with high interpretability and adaptability perform best; AI can improve through better integration of complex ecological variables.
***Species distribution modeling***	AI improves accuracy of species distribution models (SDMs) using techniques such as neural networks and ensemble learning.	Variable success in predicting population fitness and genetic diversity.	Enhancing the use of satellite imagery and real-time data for more accurate habitat predictions.
			Effective models use ensemble techniques; AI can boost accuracy with adaptive learning to incorporate real-time environmental data.
***Climate change and ecosystem dynamics***	AI to predict effects of climate change on ecosystems and species distributions.	Managing large and heterogeneous datasets; ethical concerns regarding data use.	Developing tools for real-time monitoring and better integration of satellite, ecological, and genetic data for ecosystem management.
			Models that synthesize multiple data sources show promise; AI can enhance through improved data harmonization and predictive analytics.

## References

[1] Manoj R.R.S., Latrofa M.S., Epis S., Otranto D. (2021). Wolbachia: Endosymbiont of onchocercid nematodes and their vectors. Parasites Vectors.

[2] Shoemaker D.D., Ross K.G., Keller L., Vargo E.L., Werren J.H. (2000). Wolbachia infections in native and introduced populations of fire ants (*Solenopsis* spp.). Insect Mol. Biol..

[3] Le Clec’h W., Chevalier F.D., Genty L., Bertaux J., Bouchon D., Sicard M. (2013). Cannibalism and predation as paths for horizontal passage of Wolbachia between terrestrial isopods. PLoS ONE.

[4] Yan Z.-C., Qi L.-D., Ji H.-L., Wang X.-X., Hong X.-Y., Li Y.-X. (2024). Frequent intertrophic transmission of Wolbachia by parasitism but not predation. eLife.

[5] Li S.-J., Ahmed M.Z., Lv N., Shi P.-Q., Wang X.-M., Huang J.-L., Qiu B.-L. (2017). Plantmediated horizontal transmission of Wolbachia between whiteflies. ISME J..

[6] Zimmermann B.L., Cardoso G.M., Bouchon D., Pezzi P.H., Palaoro A.V., Araujo P.B. (2021). Supergroup F Wolbachia in terrestrial isopods: Horizontal transmission from termites?. Evol. Ecol..

[7] Tseng S.-P., Hsu P.-W., Lee C.-C., Wetterer J.K., Hugel S., Wu L.-H., Lee C.-Y., Yoshimura T., Yang C.-C.S. (2020). Evidence for Com-mon Horizontal Transmission of Wolbachia among Ants and Ant Crickets: Kleptoparasitism Added to the List. Microorganisms.

[8] Audsley M.D., Seleznev A., Joubert D.A., Woolfit M., O’Neill S.L., McGraw E.A. (2018). Wolbachia infection alters the relative abundance of resident bacteria in adult Aedes aegypti mosquitoes, but not larvae. Mol. Ecol..

[9] Wiwatanaratanabutr I., Zhang C. (2016). Wolbachia infections in mosquitoes and their predators inhabiting rice field communities in Thailand and China. Acta Trop..

[10] Giesen C., Roche J., Redondo-Bravo L., Ruiz-Huerta C., Gomez-Barroso D., Benito A., Herrador Z. (2020). The impact of climate change on mosquito-borne diseases in Africa. Pathog. Glob. Health.

[11] Wang X., Xiong X., Cao W., Zhang C., Werren J.H., Wang X. (2019). Genome Assembly of the A-Group Wolbachia in Nasonia oneida Us-ing Linked-Reads Technology. Genome Biol. Evol..

[12] Taylor M.J., Bordenstein S.R., Slatko B. (2018). Microbe Profile: Wolbachia: A sex selector, a viral protector and a tar-get to treat filarial nematodes. Microbiology.

[13] Zug R., Hammerstein P. (2012). Still a host of hosts for Wolbachia: Analysis of recent data suggests that 40% of terrestrial arthropod species are infected. PLoS ONE.

[14] Hilgenboecker K., Hammerstein P., Schlattmann P., Telschow A., Werren J.H. (2008). How many species are infected with Wolbach-ia?—A statistical analysis of current data. FEMS Microbiol. Lett..

[15] Hertig M., Wolbach S.B. (1924). Studies on Rickettsia-like microorganisms in insects. J. Med. Res..

[16] Werren J.H., Hurst G.D., Zhang W., Breeuwer J.A., Stouthamer R., Majerus M.E. (1994). Rickettsial relative associate with male-killing in the ladybird beetle (*Adalia bipunctata*). J. Bacteriol..

[17] Hoffmann A.A., Ross P.A., Rašić G. (2015). Wolbachia strains for disease control: Ecological and evolutionary consid-era-tions. Evol. Appl..

[18] Ahmed M.Z., Li S.J., Xue X., Yin X.J., Ren S.X., Jiggins F.M., Greeff J.M., Qiu B.L. (2015). The intracellular bacterium Wolbachia uses parasitoid wasps as phoretic vectors for efficient horizontal transmission. PLoS Pathog..

[19] Jiggins F.M., Hurst G.D. (2011). Microbiology. Rapid insect evolution by symbiont transfer. Science.

[20] O’Neill S.L., Giordano R., Colbert A.M., Karr T.L., Robertson H.M. (1992). 16S rRNA phylogenetic analysis of the bacterial en-do-symbionts associated with cytoplasmic incompatibility in insects. Proc. Natl. Acad. Sci. USA.

[21] Werren J.H., Zhang W., Guo L.R. (1995). Evolution and phylogeny of Wolbachia: Reproductive parasites of arthropods. Proc. R. Soc. Lond. B Biol. Sci..

[22] Vavre F., Fleury F., Lepetit D., Fouillet P., Bouletreau M. (1999). Phylogenetic evidence for horizontal transmission of Wolbachia in host-parasitoid associations. Mol. Biol. Evol..

[23] Hancock P.A., White V.L., Ritchie S.A., Hoffmann A.A., Godfray H.C.J. (2016). Predicting Wolbachia invasion dynamics in Aedes aegypti populations using models of density-dependent demographic traits. BMC Biol..

[24] Laven H. (1967). Eradication of Culex pipiens fatigans through cytoplasmic incompatibility. Nature.

[25] Curtis C.F., Adak T. (1974). Population replacement in Culex fatigens by means of cytoplasmic incompatibility. Laboratory experiments with non-overlapping generations. Bull. World Health Organ..

[26] Johnson K.N. (2015). The Impact of Wolbachia on Virus Infection in Mosquitoes. Viruses.

[27] Stouthamer R., Breeuwer J.A., Hurst G.D. (1999). Wolbachia pipientis: Microbial manipulator of arthropod reproduction. Annu. Rev. Microbiol..

[28] Dodson B.L., Pujhari S., Brustolin M., Metz H.C., Rasgon J.L. (2024). Variable effects of transient Wolbachia infections on alphaviruses in Aedes aegypti. PLoS Negl. Trop. Dis..

[29] Tabachnick W.J. (2013). Nature, nurture and evolution of intra-species variation in mosquito arbovirus transmission competence. Int. J. Environ. Res. Public Health.

[30] Lewis J., Gallichotte E.N., Randall J., Glass A., Foy B.D., Ebel G.D., Kading R.C. (2023). Intrinsic factors driving mosquito vector competence and viral evolution: A review. Front. Cell. Infect. Microbiol..

[31] Gómez M., Martinez D., Muñoz M., Ramírez J.D. (2022). Aedes aegypti and Ae. albopictus microbiome/virome: New strategies for controlling arboviral transmission?. Parasites Vectors.

[32] Kaavya K., Tharakan J., Joshi C.O., Aneesh E.M. (2022). Role of vertically transmitted viral and bacterial endosymbionts of Aedes mosquitoes. Does Paratransgenesis influence vector-borne disease control?. Symbiosis.

[33] Tasaka K., Hamashima Y. (1978). Studies on rickettsia-like body in Kawasaki disease. Attempts of the isolation and characterization. Acta Pathol. Jpn..

[34] Souza-Neto J.A., Powell J.R., Bonizzoni M. (2019). Aedes aegypti vector competence studies: A review. Infect. Genet. Evol..

[35] Damasceno-Caldeira R., Nunes-Neto J.P., Aragão C.F., Freitas M.N.O., Ferreira M.S., Castro P.H.G., Dias D.D., Araújo P.A.D.S., Brandão R.C.F., Nunes B.T.D. (2023). Vector Competence of *Aedes albopictus* for Yellow Fever Virus: Risk of Reemergence of Urban Yellow Fever in Brazil. Viruses.

[36] Richard V., Paoaafaite T., Cao-Lormeau V.M. (2016). Vector Competence of French Polynesian Aedes aegypti and Aedes polynesiensis for Zika Virus. PLoS Negl. Trop. Dis..

[37] Schulz C., Becker S.C. (2018). Mosquitoes as Arbovirus Vectors: From Species Identification to Vector Competence. Mosq. Borne Dis..

[38] Kay B.H., Fanning I.D., Carley J.G. (1982). Vector competence of Culex pipiens quinquefasciatus for Murray Valley encephalitis, Kunjin, and Ross River viruses from Australia. Am. J. Trop. Med. Hyg..

[39] Mutsaers M., Engdahl C.S., Wilkman L., Ahlm C., Evander M., Lwande O.W. (2023). Vector competence of Anopheles stephensi for O’nyong-nyong virus: A risk for global virus spread. Parasites Vectors.

[40] Baimai V., O’Neill S.L. (2000). Distribution and diversity of Wolbachia infections in Southeast Asian mosquitoes (Diptera: Cu-licidae). J. Med. Entomol..

[41] Ricci I., Cancrini G., Gabrielli S., D’Amelio S., Favi G. (2002). Searching for Wolbachia (Rickettsiales: Rickettsiaceae) in mosquitoes (Diptera: Culicidae): Large polymerase chain reaction survey and new identifications. J. Med. Entomol..

[42] Rasgon J.L., Scott T.W. (2004). An initial survey for Wolbachia (Rickettsiales: Rickettsiaceae) infections in selected California mosquitoes (Diptera: Culicidae). J. Med. Entomol..

[43] Gnankine O., Dabiré R.K. (2024). Natural occurrence of Wolbachia in *Anopheles* sp. and Aedes aegypti populations could compromise the success of vector control strategies. Front. Trop. Dis..

[44] O’Connor L., Plichart C., Sang A.C., Brelsfoard C.L., Bossin H.C., Dobson S.L. (2012). Open release of male mosquitoes in-fected with a Wolbachia biopesticide: Field performance and infection containment. PLoS Negl. Trop. Dis..

[45] Rašić G., Endersby N.M., Williams C., Hoffmann A.A. (2014). Using Wolbachia-based releases for suppression of Aedes mosquitoes: Insights from genetic data and population simulations. Ecol. Appl..

[46] Teixeira L., Ferreira A., Ashburner M. (2008). The bacterial symbiont Wolbachia induces resistance to RNA viral infections in Drosophila melanogaster. PLoS Biol..

[47] Kambris Z., Cook P.E., Phuc H.K., Sinkins S.P. (2009). Immune activation by life-shortening Wolbachia and reduced filarial competence in mosquitoes. Science.

[48] Moreira L.A., Iturbe-Ormaetxe I., Jeffery J.A., Lu G., Pyke A.T., Hedges L.M., Rocha B.C., Hall-Mendelin S., Day A., Riegler M. (2009). A Wolbachia symbiont in Aedes aegypti limits infection with dengue, Chikungunya, and Plasmodium. Cell.

[49] Walker T., Johnson P.H., Moreira L.A., Iturbe-Ormaetxe I., Frentiu F.D., McMeniman C.J., Leong Y.S., Dong Y., Axford J., Kriesner P. (2011). The wMel Wolbachia strain blocks dengue and invades caged Aedes aegypti populations. Nature.

[50] Edwards B., Ghedin E., Voronin D. (2023). Wolbachia interferes with Zika virus replication by hijacking cholesterol metabolism in mosquito cells. Microbiol. Spectr..

[51] Lu P., Sun Q., Fu P., Li K., Liang X., Xi Z. (2020). Wolbachia Inhibits Binding of Dengue and Zika Viruses to Mosquito Cells. Front. Microbiol..

[52] Bhattacharya T., Yan L., Crawford J.M., Zaher H., Newton I.L.G., Hardy R.W. (2022). Differential viral RNA methylation contributes to pathogen blocking in Wolbachia-colonized arthropods. PLoS Pathog..

[53] Pinto S.B., Riback T.I.S., Sylvestre G., Costa G., Peixoto J., Dias F.B.S., Tanamas S.K., Simmons C.P., Dufault S.M., Ryan P.A. (2021). Effectiveness of Wolbachia-infected mosquito deployments in reducing the incidence of dengue and other Aedes-borne diseases in Niterói, Brazil: A quasi-experimental study. PLoS Negl. Trop. Dis..

[54] Indriani C., Tanamas S.K., Khasanah U., Ansari M.R., Rubangi Tantowijoyo W., Ahmad R.A., Dufault S.M., Jewell N.P., Utarini A., Simmons C.P. (2023). Impact of randomised wmel Wolbachia deployments on notified dengue cases and insecticide fogging for dengue control in Yogyakarta City. Glob. Health Action.

[55] Werren J.H., Baldo L., Clark M.E. (2008). Wolbachia: Master manipulators of invertebrate biology. Nat. Rev. Microbiol..

[56] Chow J.Y., Geng L., Bansal S., Dickens B.S.L., Ng L.C., Hoffmann A.A., Lim J.T. (2024). Evaluating quasi-experimental approaches for estimating epidemiological efficacy of non-randomised field trials: Applications in Wolbachia interventions for dengue. BMC Med. Res. Methodol..

[57] Kaura T., Sylvia Walter N., Kaur U., Sehgal R. (2023). Different Strategies for Mosquito Control: Challenges and Alternatives.

[58] Lu H.-Z., Sui Y., Lobo N.F., Fouque F., Gao C., Lu S., Lv S., Deng S.-Q., Wang D.-Q. (2023). Challenge and opportunity for vector control strategies on key mosquito-borne diseases during the COVID-19 pandemic. Front. Public Health.

[59] Colón-González F.J., Sewe M.O., Tompkins A.M., Sjödin H., Casallas A., Rocklöv J., Caminade C., Lowe R. (2021). Projecting the risk of mosquito-borne diseases in a warmer and more populated world: A multi-model, multi-scenario intercomparison modelling study. Lancet Planet. Health.

[60] Kraemer M.U.G., Reiner R.C., Brady O.J., Messina J.P., Gilbert M., Pigott D.M., Yi D., Johnson K., Earl L., Marczak L.B. (2019). Past and future spread of the arbovirus vectors Aedes aegypti and Aedes albopictus. Nat. Microbiol..

[61] Xavier J., Alcantara L.C.J., Fonseca V., Lima M., Castro E., Fritsch H., Oliveira C., Guimarães N., Adelino T., Evaristo M. (2023). Increased interregional virus exchange and nucleotide diversity outline the ex-pansion of chikungunya virus in Brazil. Nat. Commun..

[62] Giovanetti M., Pereira L.A., Santiago G.A., Fonseca V., Mendoza M.P.G., de Oliveira C., de Moraes L., Xavier J., Tosta S., Fristch H. (2022). Emergence of Dengue Virus Serotype 2 Cosmopolitan Genotype, Brazil. Emerg. Infect. Dis..

[63] Iani F.C.d.M., Pereira F.M., de Oliveira E.C., Rodrigues J.T.N., Machado M.H., Fonseca V., Adelino T.E.R., Guimaraes N.R., Tome L.M.R., Gomez M.K.A. (2024). Rapid spatial Expansion Beyond the Amazon Basin: Oropouche Virus joins other main arboviruses in epidemic activity across the Americas. medRxiv.

[64] Giovanetti M., Pinotti F., Zanluca C., Fonseca V., Nakase T., Koishi A.C., Tscha M., Soares G., Dorl G.G., Marques A.E.M. (2023). Genomic epide-miology unveils the dynamics and spatial corridor behind the Yellow Fever virus outbreak in Southern Brazil. Sci. Adv..

[65] Giovanetti M., Faria N.R., Lourenço J., de Jesus J.G., Xavier J., Claro I.M., Kraemer M.U., Fonseca V., Dellicour S., Thézé J. (2020). Genomic and Epidemiological Surveillance of Zika Virus in the Amazon Region. Cell Rep..

[66] Giovanetti M., de Jesus J.G., de Maia M.L., Junior J., Amarante M.C., Viana P., Barreto F.K., de Cerqueira E., Santos N.P., Falcão M.B. (2018). Genetic evidence of Zika virus in mother’s breast milk and body fluids of a newborn with severe congenital defects. Clin. Microbiol. Infect..

[67] Branda F., Nakase T., Maruotti A., Scarpa F., Ciccozzi A., Romano C., Peletto S., de Filippis A.M.B., Alcantara L.C.J., Giovanetti M. (2023). Dengue virus transmission in Italy: Surveillance and epidemiological trends up to 2023. MedRxiv.

[68] Nakase T., Giovanetti M., Obolski U., Lourenço J. (2023). Global transmission suitability maps for dengue virus transmitted by Aedes aegypti from 1981 to 2019. Sci Data..

[69] Giovanetti M., Vazquez C., Lima M., Castro E., Rojas A., de la Fuente A.G., Aquino C., Cantero C., Fleitas F., Torales J. (2023). Rapid Epidemic Expansion of Chikungu-nya Virus East/Central/South African Line-age, Paraguay. Emerg. Infect. Dis..

[70] European Centre for Disease Prevention and Control (ECDC). https://www.ecdc.europa.eu/en/mosquito-borne-diseases.

[71] Ye Y.H., Carrasco A.M., Dong Y., Sgrò C.M., McGraw E.A. (2016). The Effect of Temperature on Wolbachia-Mediated Dengue Virus Blocking in Aedes aegypti. Am. J. Trop. Med. Hyg..

[72] De La Vega F.M., Chowdhury S., Moore B., Frise E., McCarthy J., Hernandez E.J., Wong T., James K., Guidugli L., Agrawal P.B. (2021). Artificial intelligence enables comprehensive genome interpretation and nomination of candidate diagnoses for rare genetic diseases. Genome Med..

[73] Seo S., Oh M., Park Y., Kim S. (2018). DeepFam: Deep learning based alignment-free method for protein family modeling and prediction. Bioinformatics.

[74] Alarcón-Zendejas A.P., Scavuzzo A., Jiménez-Ríos M.A., Álvarez-Gómez R.M., Montiel-Manríquez R., Castro-Hernández C., Jiménez-Dávila M.A., Pérez-Montiel D., González-Barrios R., Jiménez-Trejo F. (2022). The promising role of new molecu-lar biomarkers in prostate cancer: From coding and non-coding genes to artificial intelligence approaches. Prostate Cancer Prostatic Dis..

[75] Rakotonirina A., Caruzzo C., Ballan V., Kainiu M., Marin M., Colot J., Richard V., Dupont-Rouzeyrol M., Selmaoui-Folcher N., Pocquet N. (2021). Wolbachia detection in Aedes aegypti using MALDI-TOF MS coupled to artificial intelligence. Sci. Rep..

[76] Shafin K., Pesout T., Chang P.C., Nattestad M., Kolesnikov A., Goel S., Baid G., Kolmogorov M., Eizenga J.M., Miga K.H. (2021). Haplotype-aware variant calling with PEPPER-Margin-DeepVariant enables high accuracy in nanopore long-reads. Nat. Methods.

[77] Zhou J., Troyanskaya O.G. (2015). Predicting effects of noncoding variants with deep learning–based sequence model. Nat. Methods.

[78] Etebari K., Asgari S. (2014). Accuracy of microRNA discovery pipelines in non-model organisms using closely related species genomes. PLoS ONE.

[79] Dos Santos G.R., Durovni B., Saraceni V., Riback T.I., Pinto S.B., Anders K.L., Moreira L.A., Salje H. (2022). Estimating the effect of the wMel release programme on the incidence of dengue and chikungunya in Rio de Janeiro, Brazil: A spatiotemporal modelling study. Lancet Infect. Dis..

[80] Faiz Z., Javeed S., Ahmed I., Baleanu D. (2024). Numerical investigation of a fractional order Wolbachia invasive model using sto-chas-tic Bayesian neural network. Alex. Eng. J..

[81] Joshi A., Miller C. (2022). Review of machine learning techniques for mosquito control in urban environments. Int. J. Environ. Res. Public Health.

[82] Lee-Yaw J.A., McCune J.L., Pironon S., Sheth S.N. (2024). Species distribution models rarely predict the biology of real populations. Ecol. Appl..

[83] Cao Y.T., Lu Z.P., Gao X.Y., Liu M.L., Sa W., Liang J., Wang L., Yin W., Shang Q.H., Li Z.H. (2022). Maximum Entropy Modeling the Distribution Area of *Morchella* Dill. ex Pers. Species in China under Changing Climate. Biology.

[84] van Hoek A.J., Funk S., Flasche S., Quilty B.J., van Kleef E., Camacho A., Kucharski A.J. (2024). Importance of investing time and money in integrating large language model-based agents into outbreak analytics pipelines. Lancet Microbe.

[85] Ogunlade S.T., Adekunle A.I., McBryde E.S., Meehan M.T. (2022). Modelling the ecological dynamics of mosquito populations with mul-tiple co-circulating Wolbachia strains. Sci. Rep..

[86] Carvalho V.L., Long M.T. (2021). Insect-Specific Viruses: An overview and their relationship to arboviruses of concern to humans and animals. Virology.

[87] Patterson E.I., Villinger J., Muthoni J.N., Dobel-Ober L., Hughes G.L. (2020). Exploiting insect-specific viruses as a novel strategy to control vector-borne disease. Curr. Opin. Insect Sci..

[88] Aultman K.S., Walker E.D., Gifford F., Severson D.W., Beard C.B., Scott T.W. (2000). Research ethics. Managing risks of arthropod vector research. Science.

[89] Cordaux R., Gilbert C. (2017). Evolutionary Significance of Wolbachia-to-Animal Horizontal Gene Transfer: Female Sex Determination and the f Element in the Isopod Armadillidium vulgare. Genes.

[90] Yen P.S., Failloux A.B. (2020). A Review: *Wolbachia*-Based Population Replacement for Mosquito Control Shares Common Points with Genetically Modified Control Approaches. Pathogens.

[91] Hassan M., Awan F.M., Naz A., deAndrés-Galiana E.J., Alvarez O., Cernea A., Fernández-Brillet L., Fernández-Martínez J.L., Kloczkowski A. (2022). Innovations in Genomics and Big Data Analytics for Personalized Medicine and Health Care: A Review. Int. J. Mol. Sci..

[92] Pagendam D.E., Trewin B.J., Snoad N., Ritchie S.A., Hoffmann A.A., Staunton K.M., Paton C., Beebe N. (2020). Modelling the Wolbachia incompatible insect technique: Strategies for effective mosquito population elimination. BMC Biol..

[93] Ross P.A., Turelli M., Hoffmann A.A. (2019). Evolutionary Ecology of *Wolbachia* Releases for Disease Control. Annu. Rev. Genet..

[94] Ruane A.C., Vautard R., Ranasinghe R., Sillmann J., Coppola E., Arnell N., Cruz F.A., Dessai S., Iles C.E., Islam A.K.M.S. (2022). The Climatic Impact-Driver Framework for Assessment of Risk-Relevant Climate Information. Earths Future.

[95] Alowais S.A., Alghamdi S.S., Alsuhebany N., Alqahtani T., Alshaya A.I., Almohareb S.N., Aldairem A., Alrashed M., Bin Saleh K., Badreldin H.A. (2023). Revolutionizing healthcare: The role of artificial intelligence in clinical practice. BMC Med. Educ..

